# Editorial Comment on “Risk Assessment of Cytomegalovirus Reactivation After Kidney Transplantation Under a Universal Preemptive Strategy in the Era of Intravenous Immunoglobulin‐Based Desensitization Therapy”

**DOI:** 10.1111/iju.70410

**Published:** 2026-03-17

**Authors:** Tomoaki Yamanoi, Motoo Araki

**Affiliations:** ^1^ Department of Urology Okayama University Graduate School of Medicine, Dentistry and Pharmaceutical Sciences Okayama Japan

Cytomegalovirus (CMV) infection remains a significant opportunistic complication in kidney transplant recipients, with substantial implications for both short‐term morbidity and long‐term graft survival. The study by Kijima et al. addresses a clinically important question: how does CMV reactivation manifest in contemporary kidney transplant cohorts where immunologically high‐risk recipients undergo intravenous immunoglobulin (IVIG)‐based desensitization?

This study examines 395 kidney transplant recipients stratified by IVIG and rituximab (RIT). CMV antigenemia rates were numerically higher in the IVIG (+)/RIT (+) group (25.0%) than in the RIT alone (10.7%) and standard immunosuppression (15.1%) groups, though this difference was not statistically significant [1]. Notably, all CMV antigenemia cases in the intensively desensitized group developed within 90 days of transplantation, establishing a precise window for surveillance. No patients developed symptomatic CMV syndrome or tissue‐invasive disease. These findings demonstrate that structured preemptive monitoring and antigenemia‐guided intervention are effective in this challenging setting.

The multivariate analysis identifies two independent risk factors: preoperative CMV‐IgG seronegativity (OR 2.58; 95% CI 1.36–4.88; *p* = 0.004) and immunologically high‐risk status (OR 2.56; 95% CI 1.10–5.95; *p* = 0.038). These findings reinforce established risk‐stratification principles in solid‐organ transplantation [2, 3]. CMV‐seronegative recipients lack pre‐existing immunity and depend entirely on early detection through rigorous monitoring and immune reconstitution to prevent symptomatic disease. The increased risk of CMV reactivation in immunologically high‐risk patients receiving high‐dose IVIG and RIT highlights the complex interplay between antibody‐mediated immune modulation and viral containment.

A notable observation merits consideration. Peak viral loads and clinical outcomes did not differ substantially among groups, despite higher CMV antigenemia detection rates in the IVIG (+)/RIT (+) group. This dissociation suggests that intensive immunosuppression may reduce immune containment of CMV. However, it does not necessarily amplify viral replication or clinical severity when rigorous monitoring is maintained. The mechanistic basis remains incompletely understood. IVIG preparations contain anti‐CMV antibodies at varying titers. Previous reports in ABO‐incompatible transplant recipients suggest that CMV‐specific IVIG may provide immunological protection despite the occurrence of antigenemia [4]. Conversely, intense immunosuppression may transiently impair cell‐mediated immunity, which is crucial for CMV containment. The relative contributions of these mechanisms warrant further investigation.

Several clinical implications warrant consideration. The choice between preemptive monitoring and prophylactic strategies depends on the center's resources and institutional capacity. Recent evidence suggests preemptive therapy may yield lower rates of late‐onset CMV disease and reduced drug toxicities compared to universal prophylaxis, particularly in CMV‐seronegative recipients [5]; accordingly, whether targeted CMV prophylaxis might benefit CMV‐seronegative, IVIG‐desensitized recipients warrants prospective evaluation, particularly given the concentration of events within 90 days. The availability of novel antivirals with improved safety profiles offers additional flexibility in strategy optimization. As these agents become available, center‐specific protocols should balance CMV prevention with concerns about drug toxicity and resistance, considering both institutional monitoring capacity and the intensity of immunosuppression.

In conclusion, Kijima et al. demonstrate that a carefully maintained preemptive strategy effectively prevents severe CMV disease in high‐risk populations. Their data supports risk‐stratified approaches. CMV‐seronegative or immunologically complex recipients require increased surveillance. The clustering of CMV events within 90 days suggests a logical window for intervention. Future multicenter studies comparing preemptive and prophylactic strategies in IVIG‐desensitized patients and incorporating newer antivirals would clarify optimal management and inform refinement of center‐specific protocols.

## Author Contributions

**Tomoaki Yamanoi:** conceptualization; writing – original draft. **Motoo Araki:** supervision; writing – review and editing.

## Conflicts of Interest

Motoo Araki is an Editorial Board member of the International Journal of Urology and a co‐author of this article. To minimize bias, they were excluded from all editorial decision‐making related to the acceptance of this article for publication.

## Linked Articles

This article is linked to Kijima et al. papers. To view these articles, visit https://doi.org/10.1111/iju.70342.
